# Cost-effectiveness of internet-delivered cognitive behaviour therapy for body dysmorphic disorder: Results from a randomised controlled trial

**DOI:** 10.1016/j.invent.2023.100604

**Published:** 2023-01-24

**Authors:** Oskar Flygare, Erik Andersson, Gjermund Glimsdal, David Mataix-Cols, Diana Pascal, Christian Rück, Jesper Enander

**Affiliations:** a Centre for Psychiatry Research, Department of Clinical Neuroscience, Karolinska Institutet, and Stockholm Health Care Services, Region Stockholm, Sweden; b Division of Psychology, Department of Clinical Neuroscience, Karolinska Institutet, Sweden

**Keywords:** Body dysmorphic disorder, Cognitive behaviour therapy, Cost-effectiveness

## Abstract

**Objectives:**

To evaluate the cost-effectiveness of internet-delivered cognitive behaviour therapy for body dysmorphic disorder (BDD-NET).

**Design:**

Secondary cost-effectiveness analysis from a randomised controlled trial on BDD-NET versus online supportive psychotherapy.

**Setting:**

Academic medical center.

**Participants:**

Self-referred adult participants with a primary diagnosis of body dysmorphic disorder and a score of 20 or higher on the modified Yale-Brown Obsessive Compulsive Scale for BDD (*n* = 94). Patients receiving concurrent psychotropic drug treatment were included if the dose had been stable for at least two months.

**Interventions:**

Participants received either BDD-NET (*n* = 47) or online supportive psychotherapy (n = 47) for 12 weeks.

**Primary and secondary outcome measures:**

The primary outcome measures were cost-effectiveness and cost-utility from a societal perspective, using remission status from a diagnostic interview and quality-adjusted life years (QALY), respectively. Secondary outcome measures were cost-effectiveness and cost-utility from a health care perspective and the clinic's perspective.

**Results:**

Compared to supportive psychotherapy, BDD-NET produced one additional remission for an average societal cost of $4132. The cost-utility analysis showed that BDD-NET generated one QALY to an average cost of $14,319 from a societal perspective.

**Conclusions:**

BDD-NET is a cost-effective treatment for body dysmorphic disorder, compared to online supportive psychotherapy. The efficacy and cost-effectiveness of BDD-NET should next be directly compared to in-person cognitive behaviour therapy.

**Trial registration:**

NCT02010619.

## Highlights

• First cost-effectiveness evaluation of ICBT for patients with BDD
• ICBT for BDD had 85–90 % cost-effectiveness at the primary endpoint.
• Self-rated cost estimations might not capture all costs associated with treatment.

## 1 Introduction

Body dysmorphic disorder (BDD) is characterised by an intense pre-occupation with perceived flaws in appearance that are not noticeable or appear slight to others. Cognitive behaviour therapy (CBT) is an effective treatment for BDD (Veale et al., 2014; Wilhelm et al., 2014 Jan, Wilhelm et al., 2019 Apr; Harrison et al., 2016), but few patients receive this evidence-based treatment for BDD. In one online survey, only 18 % of patients with BDD had ever received CBT (Marques et al., 2011). Barriers to treatment include shame and stigma associated with BDD, the costs of treatment, and belief that a treatment would not work (Marques et al., 2011; Buhlmann, 2011). In addition, there is a shortage of specialised CBT therapists, particularly in areas far away from universities and CBT training centers (Shapiro et al., 2003). Thus, specialist CBT for BDD is inaccessible for most patients.

Internet-delivered cognitive behaviour therapy (ICBT) has been developed for a range of mental health disorders in an effort to improve access to evidence-based treatment (Andersson et al., 2019). ICBT interventions include the same content as regular CBT and differ only in the mode of delivery: the patient logs into a secure web-based platform to access treatment content and communicate with a therapist. Previous studies have shown that therapist-guided ICBT for BDD (BDD-NET) is an efficacious treatment for adults with BDD, with effects sustained up to two years after treatment (Enander et al., 2016 Feb, Enander et al., 2019 Jan; Gentile et al., 2019). An advantage of remote treatments such as BDD-NET is that they require less therapist time per patient, enabling more patients to access therapy, and reducing costs. However, the cost-effectiveness of BDD-NET or any other treatment for BDD, is currently unknown. Here, we conducted the first cost-effectiveness evaluation of BDD-NET. Specifically, we analysed cost data from our controlled trial (Enander et al., 2016) and evaluated the cost-effectiveness of BDD-NET compared to online supportive psychotherapy. We hypothesised that BDD-NET would be cost-effective from societal, health care, and the clinic's perspectives.

## 2 Methods

### 2.1 Trial design

This study reports planned secondary cost-effectiveness analyses from a randomised controlled trial comparing BDD-NET to online supportive psychotherapy (*N* = 94) (Enander et al., 2016). The study was approved by the regional ethical review board in Stockholm (2013/1773-31/4) and pre-registered at clinicaltrials.gov (NCT02010619). Recruitment, treatment, and follow-up in the original study were conducted between November 2013 and January 2015. The results are reported in accordance with the CHEERS reporting guidelines for health economic evaluations (Husereau et al., 2013).

### 2.2 Participants

Included participants had a primary diagnosis of BDD according to the Diagnostic and Statistical Manual of Mental Disorders, 5th edition (DSM-5; American Psychiatric Association, 2013), were at least 18 years old, had access to the internet, and a score of 20 or above on the modified Yale-Brown obsessive-compulsive scale for BDD (BDD-YBOCS; Phillips et al., 1997). Participants who were taking psychotropic drugs were asked to keep their dose stable during the study period. Exclusion criteria were initiation or changes in psychotropic drug treatment within two months before enrolment, having completed CBT for BDD within the past 12 months, ongoing psychological treatment, current substance dependence, bipolar disorder or psychosis, acute suicidal ideation, or a severe personality disorder that could jeopardise participation in treatment (such as borderline personality disorder with self harm).

### 2.3 Interventions

#### 2.3.1 BDD-NET

BDD-NET was delivered through a secure online platform hosted at a dedicated hospital server with encrypted traffic. The treatment was based on a validated CBT protocol for BDD (Enander et al., 2014), contained eight text-based modules accompanied by worksheets and homework assignments, and lasted 12 weeks. Participants were asynchronously supported by a therapist through a built-in email system on the BDD-NET webpage and could log in to send emails at any time. Therapists would then review and respond to messages within 36 h on weekdays.

The therapists involved were four clinical psychology students with basic clinical training but no prior experience of treating BDD. They received weekly supervision sessions with an experienced clinician (author JE) who also monitored messages on the platform to ensure treatment integrity and adherence to protocol.

#### 2.3.2 Online supportive psychotherapy

Participants receiving online supportive psychotherapy had access to the email system on the BDD-NET webpage and received support in a similar way to BDD-NET. The therapists used counselling techniques such as reflecting, empathising and summarising in order to provide the same level of caregiver attention as BDD-NET. Notably, the supportive therapy did not include core parts of BDD-NET such as exposure with response prevention. For more detailed descriptions of the interventions, please see the main outcome paper (Enander et al., 2016).

### 2.4 Measures

Outcomes were recorded at baseline, post-treatment (3 months after baseline) and follow-up (6 months after baseline). Self-rated outcome measures were administered via the online treatment platform, and clinician-rated outcome measures were administered via telephone.

#### 2.4.1 Clinical outcomes

The cost-effectiveness analyses were based on remission status and quality-adjusted life years (QALYs). Remission was defined as no longer meeting diagnostic criteria for body dysmorphic disorder in a structured interview with a clinician blinded to treatment allocation. QALYs were estimated based on the self-rated EuroQol (EQ-5D) which is divided into five health domains: mobility, self-care, pain/discomfort, daily activities, and anxiety/depression. An index QALY score is then calculated ranging from 0 (dead) to 1 (perfect health) (Rabin and de Charro, 2001).

#### 2.4.2 Health economic measures

We used the Trimbos/iMTA questionnaire for costs associated with psychiatric illness (TIC—P) to gather information on self-rated costs in the past month (Hakkaart-van Roijen et al., 2002). The TIC-P is feasible to use in a psychiatric context and has shown high test-retest reliability and agreement with registry-based estimates (Bouwmans et al., 2013). Costs were analysed from a societal perspective by including all direct and indirect, medical and non-medical costs (e.g., productivity losses, sick leave) regardless of whether they were related to the BDD or not. From a health care perspective, costs directly associated with treatment and costs from other health care visits and medications were included. Finally, costs from the clinic's perspective included only the cost of providing treatment (e.g., therapist salary). Costs related to the development of the ICBT protocol, server costs and licensing costs were not included. Costs for medications were calculated from market prices, and health care visit costs were based on tariffs from official listings for the Swedish health care system (see Supplemental materials). Productivity losses were estimated using the human capital approach and based on the mean gross earning in Sweden in 2014 (hourly tariff of 188 SEK), and domestic work cutback was estimated using the hourly tariff of €14 (Kanters et al., 2017). The cost of unemployment was fixed at 24000 SEK ($3498) which is 80 % of a monthly salary with the mean gross earning. The TIC-P was administered at pre-treatment, post-treatment and follow-up with a recall period of 4 weeks, e.g. “how many days of sick leave did you have in the past 4 weeks?”. Costs were summarised in Swedish Crowns, extrapolated to 12 weeks in order to cover the full duration between assessments, and converted to US dollars based on purchasing power parities for the year 2014 where 6.861 SEK paid one USD.

### 2.5 Statistical analyses

All analyses were intention-to-treat including all randomised participants with the assumption that data were missing at random. Costs from the three perspectives (societal, health care, clinic) were analysed with remission status and QALY change from pre-treatment to post-treatment and follow-up as outcomes, respectively. Mixed-effects linear regression for repeated measures were used for outcomes and costs, with fixed effects of group and time, their interaction, as well as a random intercept term (see Table A2 in the Supplemental materials for details). Missing values were estimated using maximum likelihood estimation in the mixed-effects models, and non-parametric bootstrapping (1000 replications) was used to estimate group differences which were based on the group ∗ time interaction effects. The “net benefit” approach was used by estimating the probability of cost-effectiveness under different societal willingness-to-pay scenarios by calculating the proportion of bootstrap estimates below the willingness-to-pay thresholds (Drummond et al., 2005). Standardised Cohen's *d* effect sizes were estimated for both within-group and between-group changes based on the least-squares means from the models, using the residual standard deviation of random effects as sigma (Russell, 2020). We used R version 4.0.2 (R Core Team, 2020) for all analyses and the analytical code is available on the Open Science Framework https://doi.org/10.17605/OSF.IO/DC69E.

## 3 Results

### 3.1 Completion rates and participant characteristics

**Fig. 1:**
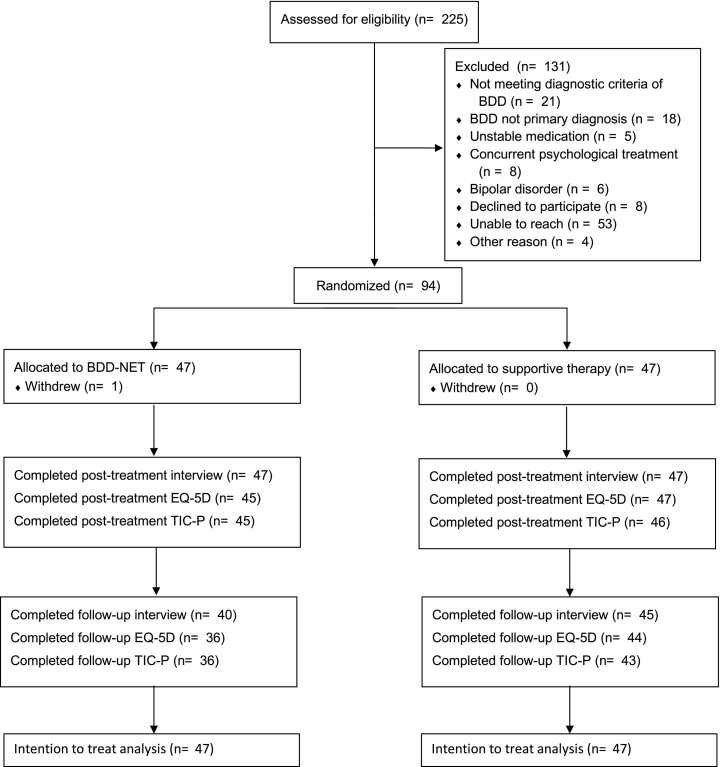
Overview of participant flow and completion of measures. Abbreviations: BDD-NET, internet-delivered therapist-guided cognitive behaviour therapy for body dysmorphic disorder; EQ-5D, EuroQol 5-Dimensions; TIC—P, Trimbos questionnaire for costs associated with psychiatric illness.

A majority of the participants were female (*n* = 80 (85 %)) and the average age of the participants was 32.6 (SD = 11.97). See Table 1 for demographic and baseline assessment information for both treatment groups. All 94 participants completed the post-treatment diagnostic interview, and at follow-up, 85 (90 %) participants completed the diagnostic interview (BDD-NET: 40/47 = 85 %, supportive therapy: 45/47 = 96 %). Completion rates for EQ-5D were similar, with 92 (98 %) assessments (BDD-NET: 45/47 = 96 %, supportive therapy: 47/47 = 100 %) at post treatment, and 80 (85 %) assessments completed at follow-up (BDD-NET: 36/47 = 77 %, supportive therapy: 44/47 = 94 %). Completion rates for the TIC-P cost assessment were high with 91 (97 %) responses at post treatment (BDD-NET: 45/47 = 96 %, supportive therapy: 46/47 = 98 %) and 79 (84 %) responses at follow-up (BDD-NET: 36/47 = 77 %, supportive therapy: 43/47 = 91 %). Fig. 1 shows participant flow through the different assessment points.

**Table 1:** Sample characteristics.

	Online supportive therapy	BDD-NET
n	47	47
Female, n (%)	41 (87 %)	39 (83 %)
Age (mean (SD))	31.32 (10.12)	33.87 (13.55)
Education, n (%)		
Primary school	6 (13 %)	4 (9 %)
High school	23 (49 %)	31 (66 %)
College/university	17 (36 %)	11 (23 %)
Doctorate	1 (2 %)	1 (2 %)
SSRI, n (%)	8 (17 %)	5 (11 %)
Pre-treatment BDD-YBOCS (mean (SD))	28.51 (4.55)	29.13 (5.02)

### 3.2 Clinical efficacy

The proportion of remitters at post-treatment, i.e. participants that no longer met diagnostic criteria according to the structured interview, was higher in the BDD-NET group compared to supportive psychotherapy at post-treatment (OR = 3.07, SE = 1.06, *p* = .004). At follow-up, results were similar with a higher proportion of remitters in the BDD-NET group (OR = 1.92, SE = 0.62, *p* = .002).

There was a small increase in EQ-5D QALY estimates from pre-treatment to post-treatment in the BDD-NET group (*d* = 0.08 [95 % CI −0.34 to 0.5]) and a medium-sized decrease in the supportive therapy group (*d* = −0.39 [95 % CI −0.8 to 0.03]). Within-group effect sizes from pre-treatment to follow-up were *d* = 0.46 [95 % CI 0 to 0.91] for BDD-NET and *d* = −0.41 [95 % CI −0.83 to 0.01] for supportive therapy. The between-group effect sizes were small at post-treatment (*d* = 0.29 [95 % CI −0.22 to 0.8]) and medium at follow-up (*d* = 0.69 [95 % CI 0.15 to 1.24]). See Table 2 for descriptive statistics at each time-point.

**Table 2:** Clinical outcomes and cost categories over time.

	Online supportive therapy	BDD-NET
Rates of remission		
Pre-treatment	N/A	N/A
Post-treatment	2 % (0 % to 14 %)	32 % (20 % to 46 %)
Follow-up	9 % (3 % to 21 %)	40 % (26 % to 56 %)
EQ-5D QALY estimate		
Pre-treatment	0.75 (0.68 to 0.81)	0.71 (0.65 to 0.78)
Post-treatment	0.67 (0.61 to 0.74)	0.73 (0.66 to 0.8)
Follow-up	0.67 (0.6 to 0.74)	0.8 (0.72 to 0.87)
Clinic's perspective costs		
Pre-treatment	N/A	N/A
Post-treatment	$461 ($332 to $590)	$946 ($817 to $1075)
Follow-up	N/A	N/A
Health care perspective costs		
Pre-treatment	$1517 ($930 to $2104)	$1262 ($674 to $1848)
Post-treatment	$1462 ($870 to $2054)	$2243 ($1646 to $2840)
Follow-up	$1193 ($587 to $1799)	$2346 ($1700 to $2993)
Societal perspective costs		
Pre-treatment	$4354 ($2756 to $5952)	$4657 ($3059 to $6255)
Post-treatment	$4052 ($2440 to $5663)	$5585 ($3960 to $7211)
Follow-up	$3808 ($2158 to $5459)	$5199 ($3437 to $6962)

Categories are additive; the health care perspective includes costs from the clinic's perspective and the societal perspective includes both health care and clinic costs. See Supplemental materials for details on specific costs included in each perspective.

Abbreviations: BDD-NET, internet-delivered cognitive behaviour therapy for body dysmorphic disorder; EQ-5D, EuroQol 5-dimensions; QALY, Quality-Adjusted Life Year.

### 3.3 Costs

Costs from the societal perspective (e.g., including all direct, indirect, medical and non-medical costs) increased in the BDD-NET group (*b* = $928) and decreased in the supportive therapy group (*b* = $-302) over the course of treatment, and decreased in both groups between post-treatment and follow-up (BDD-NET: *b* = $-386, supportive therapy: *b* = $-243). Similarly, when looking at costs from a health care perspective (e.g., direct medical costs from visits and medications), costs from pre- to post-treatment increased in BDD-NET (*b* = $982) and were stable in supportive therapy (*b* = $-55). From post-treatment to follow-up, costs were stable (BDD-NET: *b* = $103) or decreased slightly (supportive therapy: *b* = $-269). See Table 2 for a summary of costs at each time-point. Detailed descriptions of costs for all sub-categories are shown in the Supplemental materials.

The cost of providing treatment from the clinic's perspective included the salary cost of therapists and was estimated to $946 for BDD-NET and $461 for supportive therapy. This cost corresponds to an average time of 15.7 min per patient and week for BDD-NET and 7.7 min for supportive therapy.

### 3.4 Cost-effectiveness and cost-utility

#### 3.4.1 The societal perspective

**Fig. 2:**
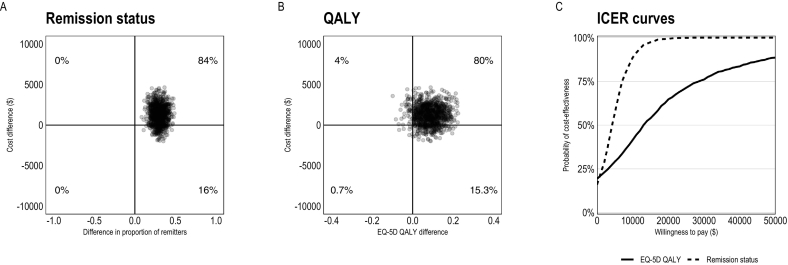
Pre-treatment to post-treatment cost-effectiveness of BDD-NET compared to supportive therapy. Cost-effectiveness planes for BDD remission rates (panel A), QALYs (panel B), and acceptability curves (panel C). The x-axis in panels A and B displays between-group differences in outcomes, with positive values indicating more improvement in the BDD-NET group compared to supportive therapy. Costs are from a societal perspective and based on the TIC-P questionnaire using Swedish health care tariff listings and national statistics. Abbreviations: BDD-NET, internet-delivered cognitive behaviour therapy for body dysmorphic disorder; ICER, incremental cost-effectiveness ratio; QALYs, Quality-adjusted life years.

**Fig. 3:**
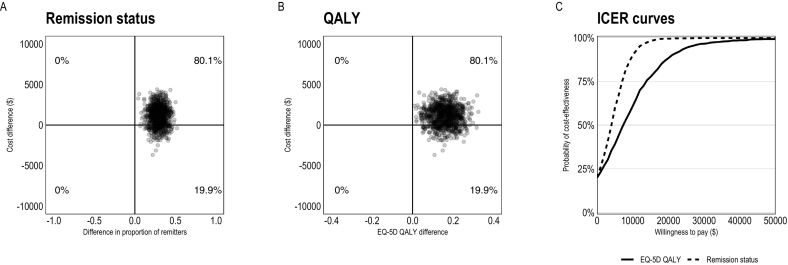
Pre-treatment to follow-up cost-effectiveness of BDD-NET compared to supportive therapy. Cost-effectiveness planes for BDD remission rates (panel A), QALYs (panel B), and acceptability curves (panel C). The x-axis in panels A and B displays between-group differences in outcomes, with positive values indicating more improvement in the BDD-NET group compared to supportive therapy. Costs are from a societal perspective and based on the TIC-P questionnaire using Swedish health care tariff listings and national statistics. Abbreviations: BDD-NET, internet-delivered cognitive behaviour therapy for body dysmorphic disorder; ICER, incremental cost-effectiveness ratio; QALYs, Quality-adjusted life years.

The cost-effectiveness of BDD-NET versus the supportive therapy control treatment was investigated up until the 3-month controlled follow-up, after which the control group was offered BDD-NET. To estimate cost-effectiveness, between-group differences in costs were compared to between-group differences in the proportion of participants in remission. The cost-effectiveness ICER at post-treatment was 1231/0.3 = $4132 per case in remission. At follow-up, the corresponding numbers were 1088/0.29 = $3731 per case in remission. In short, this means that BDD-NET was cost-effective from a societal perspective given that society is willing to pay up to $4132 per case in remission after treatment. A majority of ICERs from bootstrapped samples were located in the northeast quadrant, confirming higher efficacy and costs of BDD-NET compared to online supportive therapy (Figs. 2A, 3A).

Cost-utility analyses (analyses of QALYs in relation to costs) indicated that the societal cost for one additional QALY gain from BDD-NET versus supportive therapy was 1231/0.09 = $14,319 at post-treatment, and 1088/0.16 = $6784 at follow-up. BDD-NET reached 75 % cost-utility at a societal willingness to pay of $29,000 at post-treatment and $14,000 at follow-up (Figs. 2C, 3C). Further, BDD-NET showed a cost utility of 85 %–90 % for thresholds of £25,000–35,000 ($41,000–$58,000) (Rawlins and Culyer, 2004) and 89 % cost-utility at the commonly used $50,000 threshold (Grosse, 2008) at post-treatment, and a cost-utility of 98–99 % at these thresholds at follow-up. Again, the majority of ICERs from bootstrapped samples were located in the northeast quadrant of the cost-effectiveness plane (Figs. 2B, 3B).

#### 3.4.2 The health care perspective

From the health care perspective, which includes costs of health care and medications (but excludes non-medical or indirect costs), the cost-effectiveness of BDD-NET at post treatment was 1037/0.3 = $3482 per case in remission. The cost-effectiveness of BDD-NET versus supportive therapy at follow-up was 1409/0.29 = $4831. Compared to supportive therapy, BDD-NET was estimated to have an additional cost of $3482 per case in remission, and the ICERs from bootstrapped samples were located in the northeast quadrant of the cost-effectiveness plane (see Supplemental materials).

#### 3.4.3 The clinic's perspective

The clinic's perspective includes the cost of delivering the interventions (in this case, the cost of the psychologists' time). The cost-effectiveness of BDD-NET compared to supportive therapy at post-treatment was 485/0.3 = $1628 per case in remission. At follow-up, cost-effectiveness was 485/0.29 = $1662. Again, the ICERs from bootstrapped samples were located in the northeast quadrant of the cost-effectiveness plane.

#### 3.4.4 Sensitivity analyses

Sensitivity analyses were conducted in order to check the robustness of the results. First, the cost-effectiveness and cost-utility analyses were re-run with complete cases only and no missing data imputation. The results were identical compared to the main ITT analyses with the same proportion of bootstrap samples in each quadrant at both post-treatment and follow-up (see Figs. A6 and A7 in the Supplementary materials). Second, as costs for unemployment are large and can be impacted by a few individual cases, costs from the societal perspective were re-analysed excluding unemployment costs. Due to a large variation in the supportive therapy group with low costs at post treatment ($228) compared to both pre-treatment ($893) and follow-up ($1220), the differences in societal costs were smaller at post-treatment when excluding this cost category. Only 55 % of bootstrapped samples showed higher costs for BDD-NET when excluding unemployment costs compared to 84 % of samples in the full societal perspective. At follow-up, however, the results were robust to the costs of unemployment, with 80 % and 85 % of samples showing higher costs for BDD-NET when including and excluding unemployment costs, respectively (see Figs. A1 to A4 in the Supplementary materials).

## 4 Discussion

To our knowledge, this is the first cost-effectiveness study of a psychological treatment for BDD. We found that therapist-guided internet-delivered cognitive behaviour therapy for body dysmorphic disorder (BDD-NET) produced one additional remission for an average societal cost of $4132 and the cost-utility analysis showed that BDD-NET generated one additional QALY to an average cost of $14,319 from a societal perspective, compared to online supportive therapy. BDD-NET has a probability of 85 % to 90 % of being cost-effective from a societal perspective at multiple commonly used willingness-to-pay thresholds for one QALY ($41,000–$58,000). Results were similar when costs from a health care perspective and the clinic's perspective were considered.

Previous studies on the cost-effectiveness internet-delivered cognitive behaviour therapy (ICBT) for related disorders have found similar results, also using self-reported estimates of costs and resource use from the TIC—P. For example, Andersson et al. (2015) found that an internet-delivered CBT for obsessive-compulsive disorder produced an additional remission for $672 and one additional QALY for $4800 compared to online supportive therapy. ICBT for obsessive-compulsive disorder is also cost-saving compared to face-to-face treatment, saving up to $6153 from a societal perspective (Lundström et al., 2022). In cost-effectiveness analyses of ICBT for health anxiety, savings of £1244 per additional case of remission were found compared to an attention control condition (Hedman et al., 2013), and several forms of low-intensity treatments were found to be cost-effective compared to waiting list (£-134 to £416 per additional case in remission) (Axelsson et al., 2018). Similarly, ICBT for depression was associated with a cost of €1817 for each additional reliably improved participant (Warmerdam et al., 2010) and has been estimated to be cost-effective at a societal willingness to pay of £20,000 per additional QALY compared to usual care (Hollinghurst et al., 2010). Caution should be taken in directly comparing results from different studies since they may differ in the estimation of costs, outcome definitions, health care context, or comparators. For example, costs associated with developing the treatment protocol, server costs as well as licensing costs were not included in the current study, and should be considered before implementation in other health care contexts. Keeping this limitation in mind, a meta-analysis of ICBT for various conditions found that the treatments are cost-effective options by providing large clinical gains at relatively low cost (Donker et al., 2015).

Of note, the findings from this study support previous research showing markedly poor quality of life in individuals with BDD, even when compared to other psychiatric disorders (Phillips et al., 2005). For example, the EQ-5D estimate at post-treatment in the current trial was 0.73 for the group receiving BDD-NET, which is lower than pre-treatment estimates for both OCD (0.8) (Andersson et al., 2015) and health anxiety (0.83) (Axelsson et al., 2018). Thus, despite being effective in terms of symptom reduction, more work is needed to ensure that treatments for BDD provide patients with the tools necessary to improve their functioning and quality of life.

The results should be viewed in light of several limitations. First, the current study used self-reported costs from the TIC—P, which might not capture all costs associated with treatment. However, the cost estimations from the TIC-P are reliable and correspond well to costs estimated from medical registrations (Bouwmans et al., 2013). Second, BDD-NET was compared to supportive psychotherapy which is not an established treatment for BDD, however face to face supportive psychotherapy was associated with comparable gains to CBT for BDD in a recent randomised controlled trial, indicating that it is an effective treatment for some patients with BDD (Wilhelm et al., 2019). Third, participants in this study were self-referred and thus likely to be motivated to undergo treatment. This may not be representative for the whole population of patients with BDD, who may have poor insight and be less motivated to seek help for their problems. Fourth, treatment was delivered in a specialised academic setting where treating therapists received supervision by experts in BDD. If the treatment is delivered in other settings these resources may not be readily available.

Future research should directly compare the efficacy and cost-effectiveness of BDD-NET to face-to-face CBT for BDD which, along with SSRI medication, is a first line treatment for BDD. Additionally, evaluation of BDD-NET in regular psychiatric care is needed as these patients may differ in level of insight, psychiatric comorbidity and willingness to undergo psychological treatment for their BDD, compared to self-referred participants.

In conclusion, BDD-NET is a cost-effective treatment for BDD compared to online supportive psychotherapy, with an 85–90 % probability of being cost-effective at commonly used willingness-to-pay thresholds. The results have direct implications for rational decision-making in health care, where information on the cost-effectiveness of treatment alternatives can improve the distribution of limited resources, thus providing effective treatment to more patients at a minimal cost.

## CRediT authorship contribution statement

JE and CR had the original idea for the study and, with DM-C, designed the trial variables and obtained funding. JE and CR were responsible for study supervision. OF carried out the statistical analysis and drafted the manuscript, which was revised by EA, GG, DM-C, DP, CR and JE. All authors approved the final version to be submitted.

## Funding

This research received no specific grant from any funding agency in the public, commercial or not-for-profit sectors. The original clinical trial was funded through the regional agreement on medical training and clinical research (ALF) between the Region Stockholm and 10.13039/501100004047Karolinska Institutet, the 10.13039/501100004359Swedish Research Council (no. K2013-61X-22168-01-3) and the 10.13039/501100007687Swedish Society of Medicine (Söderströmska Königska sjukhemmet, no. SLS3B4451).

The funders had no part in the study design, in the collection, analysis and interpretation of data, in the writing of the report or in the decision to submit the article for publication.

## Ethical approval

The regional ethical review board in Stockholm approved the protocol (registration ID: 2013/1773-31/4). All participants gave written informed consent.

## Patient and public involvement

No patients were involved in setting the research question or the outcome measures, nor were they involved in developing plans for recruitment, design, or implementation of the study. No patients were asked to advise on interpretation or writing up of the results.

## Declaration of competing interest

The authors declare that they have no known competing financial interests or personal relationships that could have appeared to influence the work reported in this paper.

## Data Availability

The datasets analysed are not publicly available since they contain sensitive personal identifying information and data sharing was not part of the written informed consent, but are available from the corresponding author on reasonable request. Statistical code is publicly available from the Open Science Framework repository: https://doi.org/10.17605/OSF.IO/DC69E.

## Appendix A Supplementary data

Supplementary methods and results Image 1
