# Supplementary material for: Cost-effectiveness of internet-delivered cognitive behaviour therapy for body dysmorphic disorder: Results from a randomised controlled trial
Source: Internet Interv. 2023 Jan 24;31:100604. doi: 10.1016/j.invent.2023.100604 (PMC9900508; doi:10.1016/j.invent.2023.100604)
Supplement: Supplementary file 1 — Supplementary methods and results [file mmc1.docx]

Supplemental materials

*For article: Cost-effectiveness of internet-delivered cognitive behaviour therapy for body dysmorphic disorder: results from a randomized controlled trial*

*Oskar Flygare, Erik Andersson, Gjermund Glimsdal, David Mataix-Cols, Diana Pascal, Christian Rück, Jesper Enander*

1. Supplemental methods 2

1.1 Cost sources 2

2. Supplemental results 3

2.1 Detailed mixed-effects models results 3

2.2 Detailed cost breakdown 4

2.3 Cost-effectiveness planes 5

2.4 Complete-case cost-effectiveness and cost-utility analyses 8

# 1. Supplemental methods

## 1.1 Cost sources

Costs for medications were estimated based on the prices in The Dental and Pharmaceutical Benefits Agency database, which is publicly available online (https://www.tlv.se/beslut/sok-i-databasen.html). If dose or product name was not reported in TIC-P (for example responding “SSRI”), costs were estimated based on the standard dose of the most common product. Costs for alternative medications were based on market prices. Standard costs for health care visits in both outpatient and inpatient care are shown in table A.1.

The Swedish health care system is funded by taxes and patients do not pay the full cost of health care visits themselves. In 2014 there was a payment cap at 1100 SEK (~160 US dollar), after which patients did not pay for additional visits. In addition, some health care visits are always free (e.g., midwife consultations for pregnant women). Similarly, the costs of medications are subsidized and there was a payment cap for medications at 2200 SEK (~320 US dollar) in 2014. These payment caps were ignored in the cost and resource use estimations and the total costs for all visits and medications were included.

| Table A.1. Standard costs for health care visits | | |
| --- | --- | --- |
| Health service | Standard cost | Unit |
| General practitioner | $ 320 | Consultation |
| Company physician | $ 175 | Consultation |
| Psychiatrist | $ 662 | Consultation |
| Medical specialist (other) | $ 446 | Consultation |
| District nurse | $ 107 | Consultation |
| Psychotherapist, private practice | $ 146 | Consultation |
| Psychotherapist, primary care | $ 301 | Consultation |
| Psychotherapist, psychiatry | $ 477 | Consultation |
| Counselor | $ 287 | Consultation |
| Physiotherapist | $ 107 | Consultation |
| Midwife | $ 199 | Consultation |
| Alternative care | $ 87 | Consultation |
| Home care | $ 58 | Hour |
| Self-help group | $ 13 | Hour |
| Note. All costs are in 2014-06-01 US dollar, converted from the Swedish krona. Most estimates are based on official public listings in the publicly funded Swedish health care system. | | |

The cost of unemployment was estimated at 24000 SEK per month for all individuals indicating unemployment, which assumes that everyone received 80% of a full-time salary with the mean gross earning in Sweden in 2014. However, there are multiple factors that can affect this amount, for example the pre-unemployment salary, form of employment, the length of unemployment and whether the individual is a member of an unemployment insurance fund or not. As the current study did not ask in detail regarding those factors, the cost of unemployment is not individualized and care should be taken when interpreting the results.

# 2. Supplemental results

## 2.1 Detailed mixed-effects models results

The models were fit using the *lmer()* function in the *lme4* package in R, using fixed effects of group and time as well as a random intercept. The raw model output is showed below. Between-group and within-group changes over time were then estimated using the *emmeans* package and uncertainty around the estimates were calculated using non-parametric bootstrapping using the *bootstrap_model()* function from the *parameters* package.

| Table A.2. Detailed mixed-effects models output | | | | | |
| --- | --- | --- | --- | --- | --- |
| Term | *Estimate* | *SE* | *Statistic* | *df* | *p* |
| **Remission status** | | | | | |
| Intercept | 0 | 0,04 | 0 | 224,66 | p=1 |
| BDD-NET | 0 | 0,06 | 0 | 224,66 | p=1 |
| Post-treatment | 0,02 | 0,05 | 0,42 | 179,63 | p=.6728 |
| Follow-up | 0,09 | 0,05 | 1,85 | 181,26 | p=.0662 |
| BDD-NET:Post-treatment | 0,3 | 0,07 | 4,19 | 179,63 | p<.001 |
| BDD-NET:Follow-up | 0,29 | 0,07 | 3,97 | 183,51 | p<.001 |
| **EQ-5D QALY** | | | | | |
| Intercept | 0,75 | 0,03 | 22,47 | 219,95 | p<.001 |
| BDD-NET | -0,03 | 0,05 | -0,68 | 219,95 | p=.4959 |
| Post-treatment | -0,07 | 0,04 | -1,87 | 174,11 | p=.0627 |
| Follow-up | -0,08 | 0,04 | -1,95 | 176,49 | p=.0526 |
| BDD-NET:Post-treatment | 0,09 | 0,05 | 1,58 | 175,54 | p=.1159 |
| BDD-NET:Follow-up | 0,16 | 0,06 | 2,81 | 180,81 | p=.0055 |
| **Treatment costs** | | | | | |
| Intercept | 0 | 64,75 | 0 | 188 | p=1 |
| BDD-NET | 0 | 91,57 | 0 | 188 | p=1 |
| Post-treatment | 460,79 | 64,75 | 7,12 | 188 | p<.001 |
| Follow-up | 460,79 | 64,75 | 7,12 | 188 | p<.001 |
| BDD-NET:Post-treatment | 484,91 | 91,57 | 5,3 | 188 | p<.001 |
| BDD-NET:Follow-up | 484,91 | 91,57 | 5,3 | 188 | p<.001 |
| **Direct medical costs** | | | | | |
| Intercept | 1516,61 | 294,21 | 5,15 | 187,19 | p<.001 |
| BDD-NET | -255,61 | 416,08 | -0,61 | 187,19 | p=.5398 |
| Post-treatment | -54,74 | 302,9 | -0,18 | 173,97 | p=.8568 |
| Follow-up | -323,96 | 309,85 | -1,05 | 175,72 | p=.2972 |
| BDD-NET:Post-treatment | 1037,07 | 430,13 | 2,41 | 174,61 | p=.0169 |
| BDD-NET:Follow-up | 1409,45 | 452,63 | 3,11 | 178,39 | p=.0022 |
| **Societal costs** | | | | | |
| Intercept | 4354,21 | 801,16 | 5,43 | 188 | p<.001 |
| BDD-NET | 302,64 | 1133,01 | 0,27 | 188 | p=.7897 |
| Post-treatment | -302,39 | 828,49 | -0,36 | 173,7 | p=.7156 |
| Follow-up | -545,82 | 847,48 | -0,64 | 175,48 | p=.5204 |
| BDD-NET:Post-treatment | 1230,72 | 1176,48 | 1,05 | 174,35 | p=.297 |
| BDD-NET:Follow-up | 1088,4 | 1237,93 | 0,88 | 178,18 | p=.3805 |
| Abbreviations: df; degrees of freedom; EQ-5D, EuroQol 5 Dimensions; p, p-value; QALY, Quality-Adjusted Life Year; SE, standard error. | | | | | |

## 2.2 Detailed cost breakdown

In the tables below, costs from various sources in the TIC-P are shown separately. Costs for providing therapy (i.e., therapist time) are not included. Costs for health care visits are included if they were unrelated to the clinical trial, since all visits in the research study were free of charge for participants.

The health care perspective included direct medical costs for healthcare visits and medications, as well as costs for providing therapy. In the full societal perspective, costs from all categories were included.

| Table A.3. TIC-P costs at pre-treatment | | |
| --- | --- | --- |
| Pre-treatment |  |  |
|  | BDD-NET (n = 47) | Supportive psychotherapy (n = 47) |
| Direct medical costs | 1261 (1943), 389 | 1517 (2122), 331 |
| *Healthcare visits* | 1162 (1893), 304 | 1473 (2110), 321 |
| *Medication* | 99 (311), 15 | 43 (57), 9 |
| Direct non-medical costs | 6 (38), 0 | 0 (0), 0 |
| Indirect costs | 3396 (5222), 610 | 2838 (4821), 689 |
| *Unemployment* | 1340 (3540), 0 | 893 (2960), 0 |
| *Sick leave* | 1000 (3367), 0 | 608 (2284), 0 |
| *Work cutback* | 663 (2247), 0 | 808 (2231), 0 |
| *Domestic* | 393 (795), 112 | 528 (1167), 38 |
| **Gross total costs** | **4662 (6249), 2261** | **4354 (5821), 1788** |
| *Note.* Mean (SD), median. All costs are in 2014-06-01 US Dollar and extrapolated to a three-month period. | | |

| Table A.4. TIC-P costs at post-treatment | | |
| --- | --- | --- |
| Post-treatment |  |  |
|  | BDD-NET (n = 45) | Supportive psychotherapy (n = 46) |
| Direct medical costs | 1268 (2661), 2 | 1023 (1766), 115 |
| *Healthcare visits* | 1194 (2619), 0 | 969 (1769), 0 |
| *Medication* | 74 (234), 0 | 54 (202), 7 |
| Direct non-medical costs | 47 (188), 0 | 34 (171), 0 |
| Indirect costs | 3404 (4848), 658 | 2619 (4234), 712 |
| *Unemployment* | 1866 (4057), 0 | 228 (1547), 0 |
| *Sick leave* | 772 (2775), 0 | 869 (2434), 0 |
| *Work cutback* | 455 (1296), 0 | 1131 (2699), 234 |
| *Domestic* | 311 (577), 37 | 390 (731), 65 |
| **Gross total costs** | **4719 (6441), 1233** | **3677 (4781), 1647** |
| *Note.* Mean (SD), median. All costs are in 2014-06-01 US Dollar and extrapolated to a three-month period. | | |

| Table A.5. TIC-P costs at follow-up | | |
| --- | --- | --- |
| Follow-up |  |  |
|  | BDD-NET (n = 36) | Supportive psychotherapy (n = 43) |
| Direct medical costs | 1396 (2208), 588 | 714 (1345), 44 |
| *Healthcare visits* | 1296 (2161), 0 | 679 (1338), 0 |
| *Medication* | 101 (351), 7 | 35 (67), 4 |
| Direct non-medical costs | 0 (0), 0 | 0 (0), 0 |
| Indirect costs | 2941 (4480), 229 | 2767 (4389), 446 |
| *Unemployment* | 1749 (3966), 0 | 1220 (3404), 0 |
| *Sick leave* | 580 (2285), 0 | 319 (1250), 0 |
| *Work cutback* | 372 (1240), 0 | 947 (2208), 0 |
| *Domestic* | 240 (653), 2 | 282 (580), 8 |
| **Gross total costs** | **4337 (5159), 2479** | **3481 (4791), 1164** |
| *Note.* Mean (SD), median. All costs are in 2014-06-01 US Dollar and extrapolated to a three-month period. | | |

| Table A.6. Number of individuals in unemployment | | |
| --- | --- | --- |
|  | BDD-NET, n (%) | Supportive psychotherapy, n (%) |
| Pre-treatment | 6 (13%) | 4 (9%) |
| Post-treatment | 8 (18%) | 1 (2%) |
| Follow-up | 6 (17%) | 5 (12%) |

## 2.3 Cost-effectiveness planes

The supplemental cost-effectiveness planes show costs versus effect at post-treatment and follow-up from all three perspectives. In the societal perspective, all direct and indirect medical and non-medical costs are included; in the direct medical perspective, only direct medical costs are included (i.e., treatment costs, health care visits and medications); in the health organisational perspective, only the cost of providing treatment is included.

As a sensitivity analysis, cost-effectiveness planes from a societal perspective excluding unemployment are shown alongside the full societal perspective.


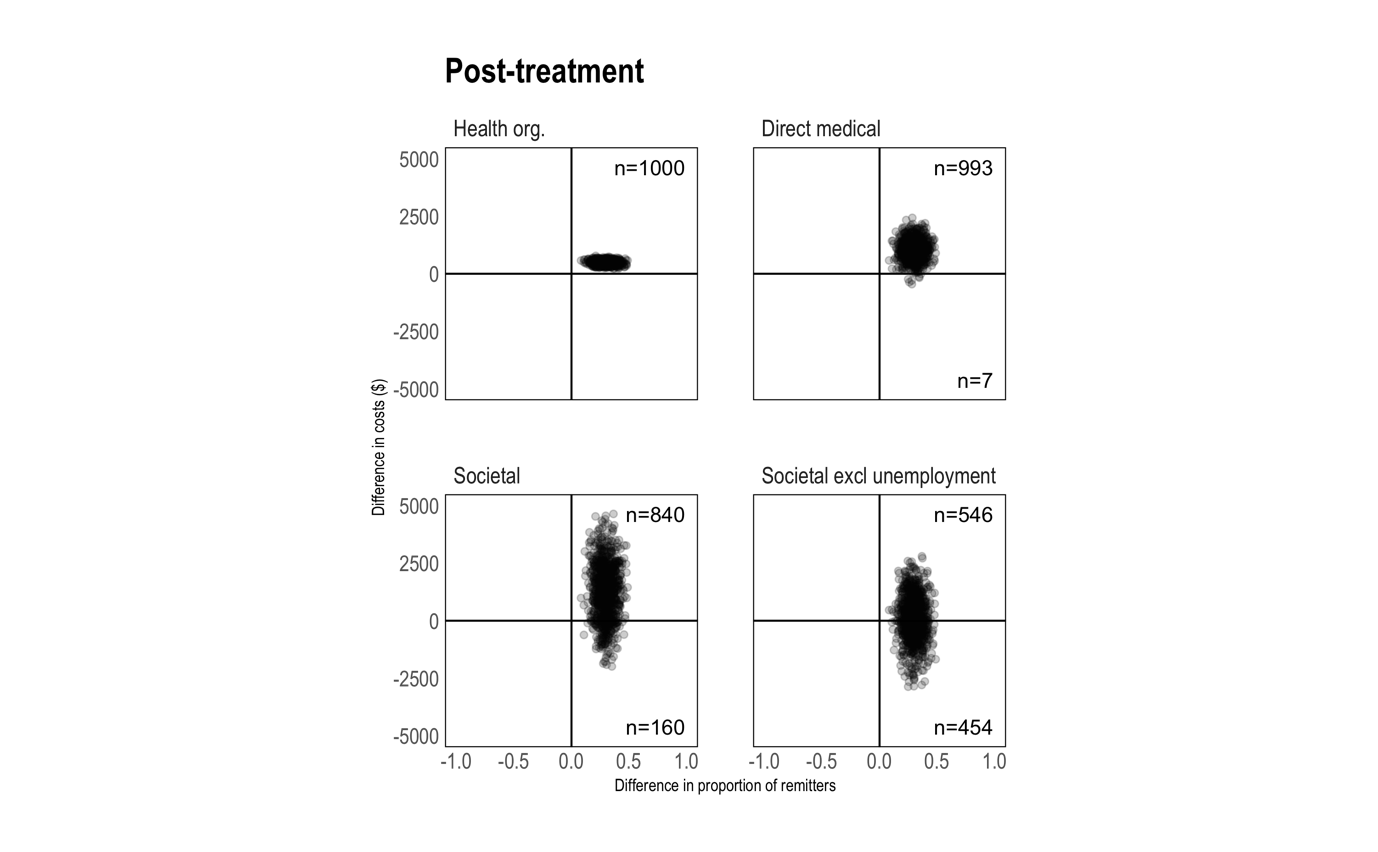


Figure A.1. Cost-effectiveness of BDD-NET versus supportive psychotherapy from pre- to post-treatment. Effect based on proportion of patients in remission after treatment.
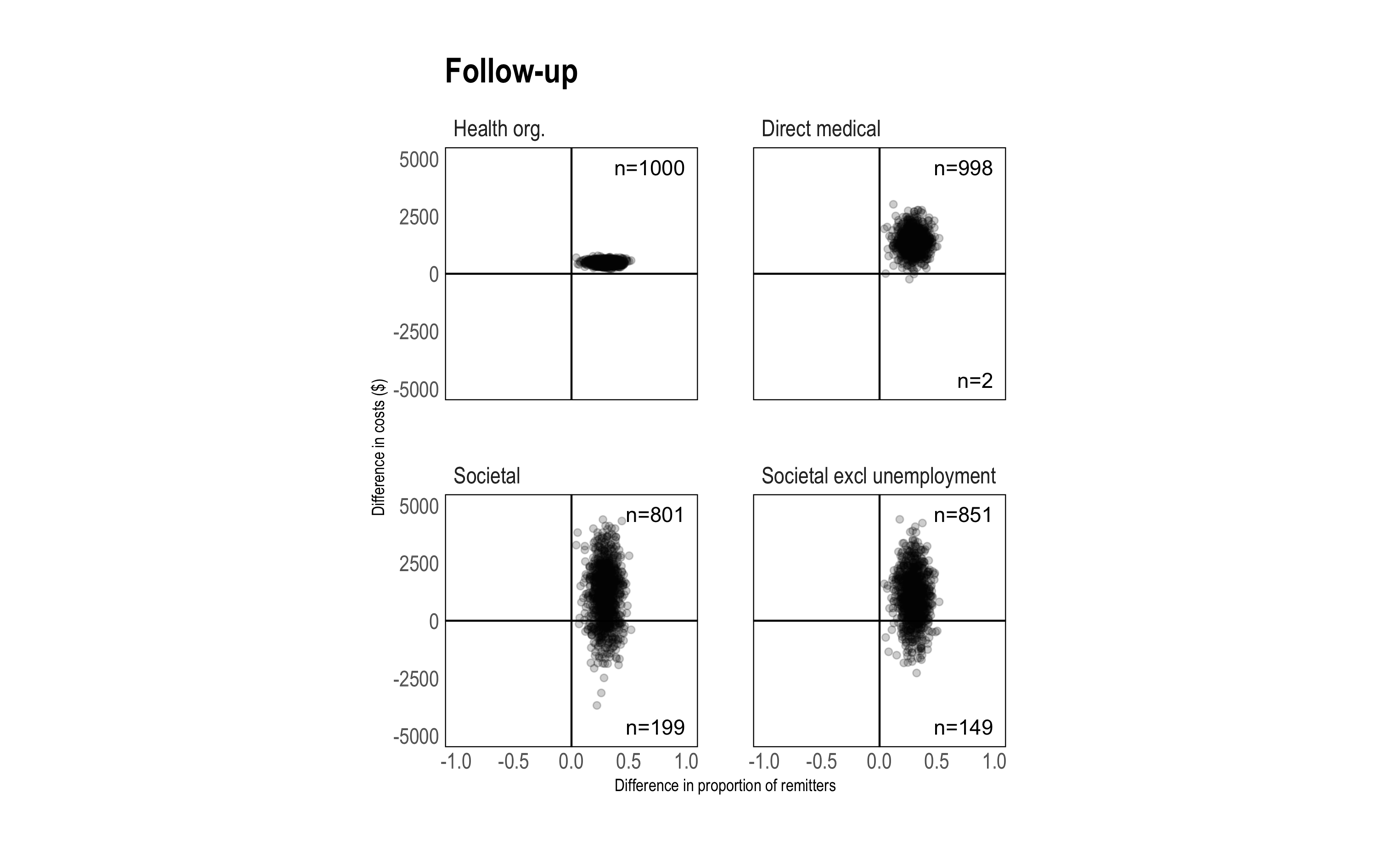
 Figure A.2. Cost-effectiveness of BDD-NET versus supportive psychotherapy from pre-treatment to follow-up. Effect based on proportion of patients in remission at follow-up.


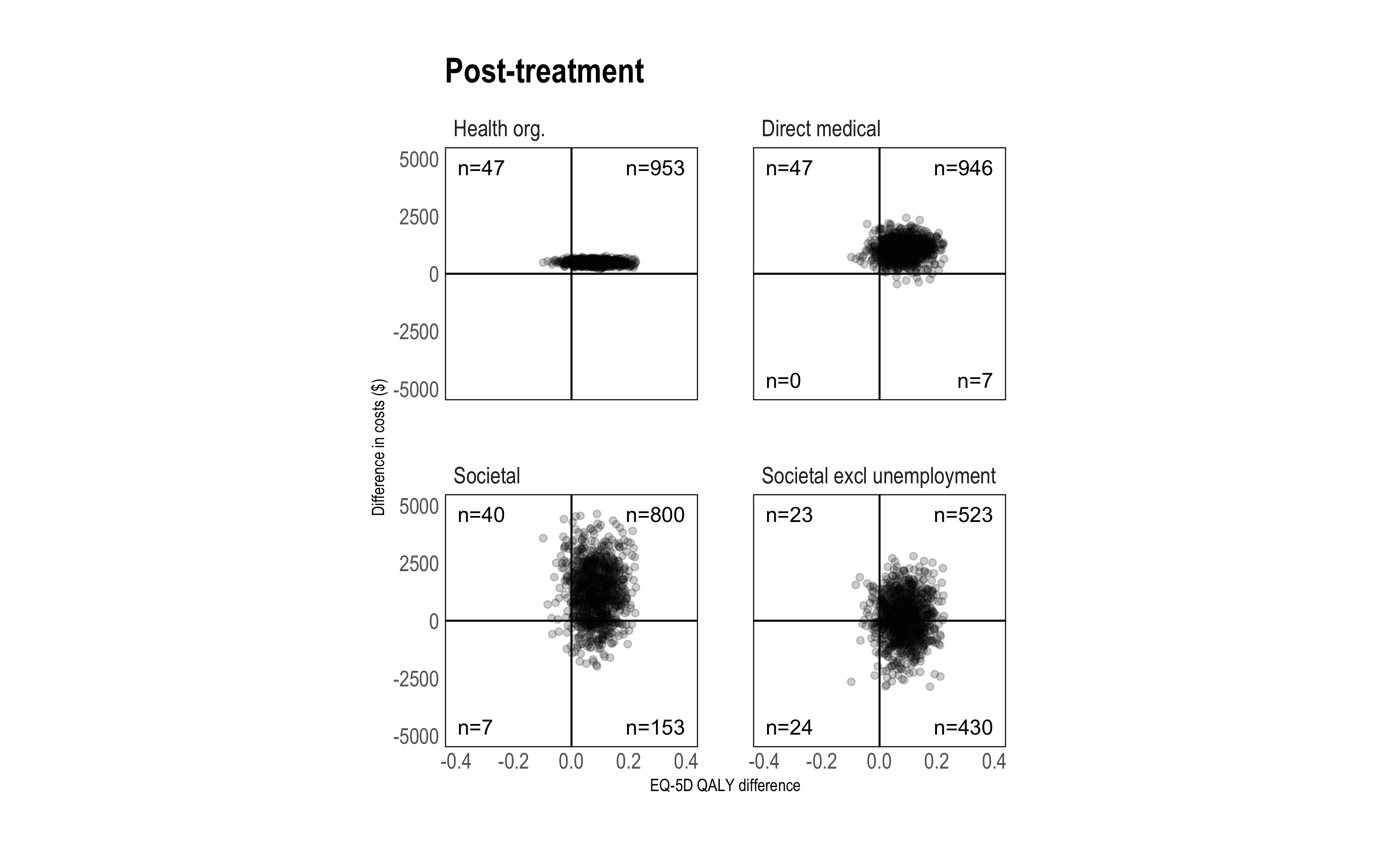


Figure A.3. Cost-utility of BDD-NET versus supportive psychotherapy from pre- to post-treatment. Effect based on quality-adjusted life year (QALY) change from pre- to post-treatment.


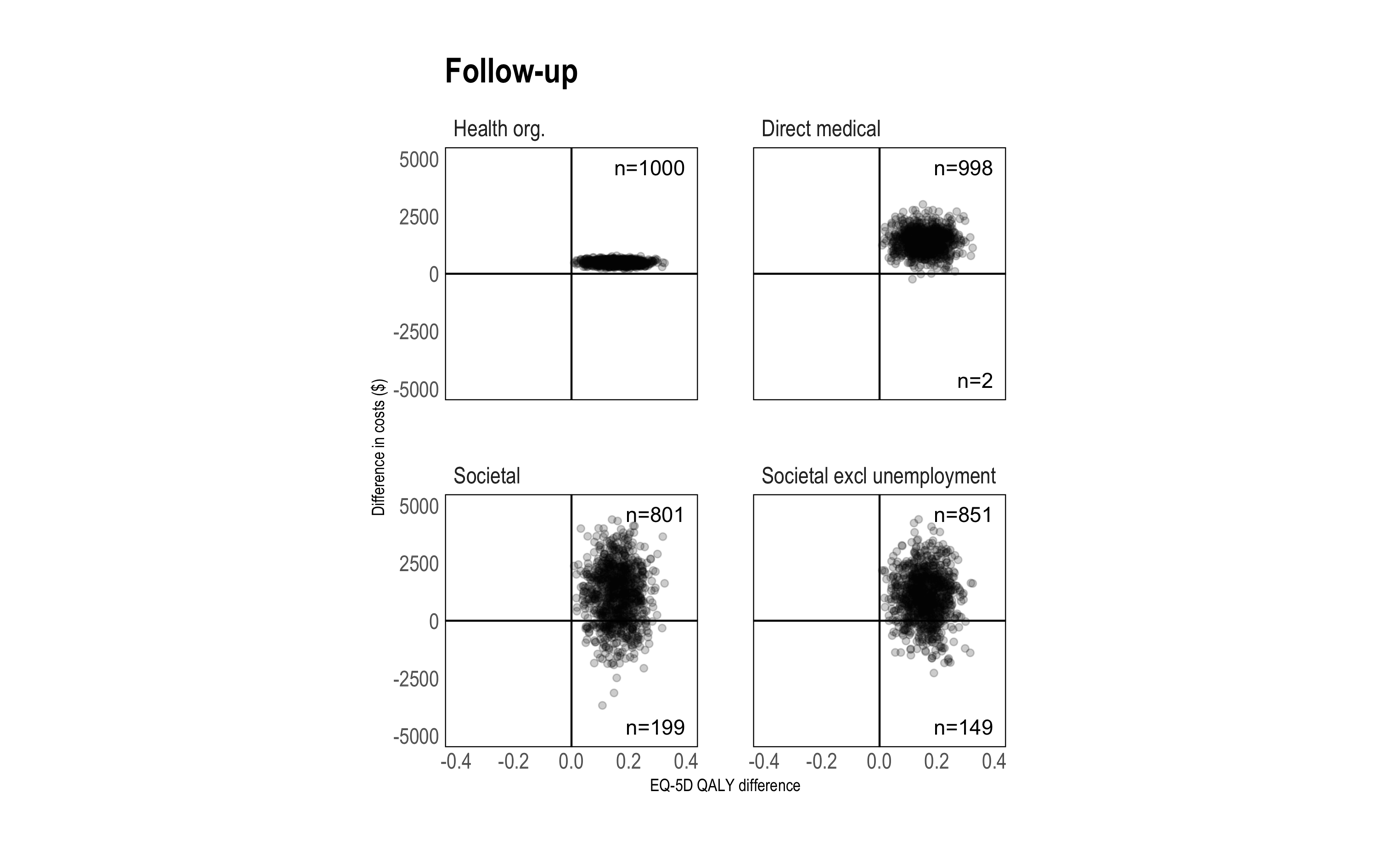


Figure A.4. Cost-utility of BDD-NET versus supportive psychotherapy from pre- to follow-up. Effect based on quality-adjusted life year (QALY) change from pre-treatment to follow-up.


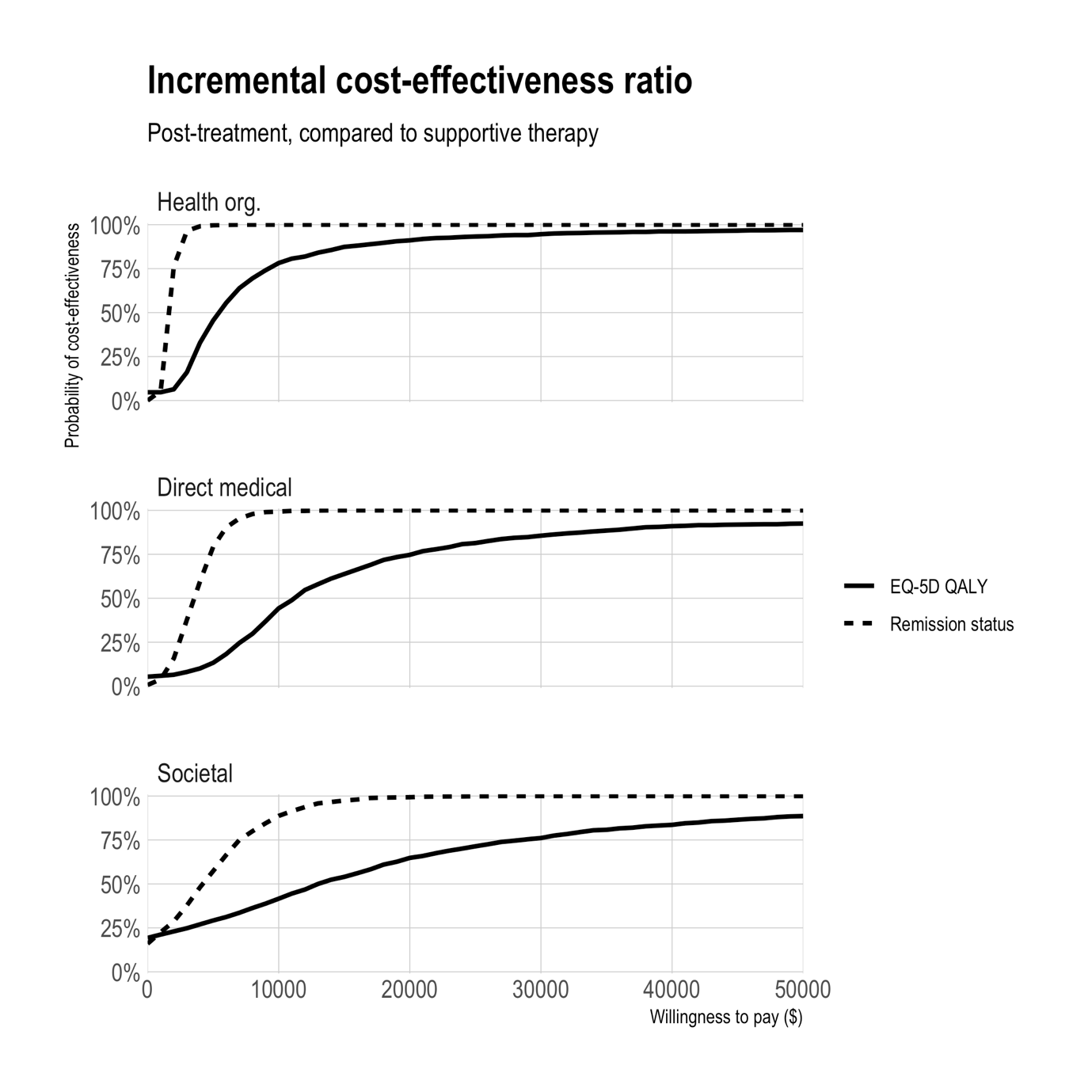


Figure A.5. Acceptability curves at post-treatment for quality-adjusted life years and remission status, with costs from different perspectives.

## 2.4 Complete-case cost-effectiveness and cost-utility analyses

Cost-effectiveness and cost-utility analyses were conducted using only participants that provided data at post-treatment and follow-up, respectively. All other modeling parameters were identical to the main analyses.

The proportion of missing data on the outcome of remission status was 0% at post-treatment and 10% at follow-up. On the EQ-5D, missing data was 2% at post-treatment and 15% at follow-up. TIC-P missingness was 3% at post-treatment and 16% at follow-up.


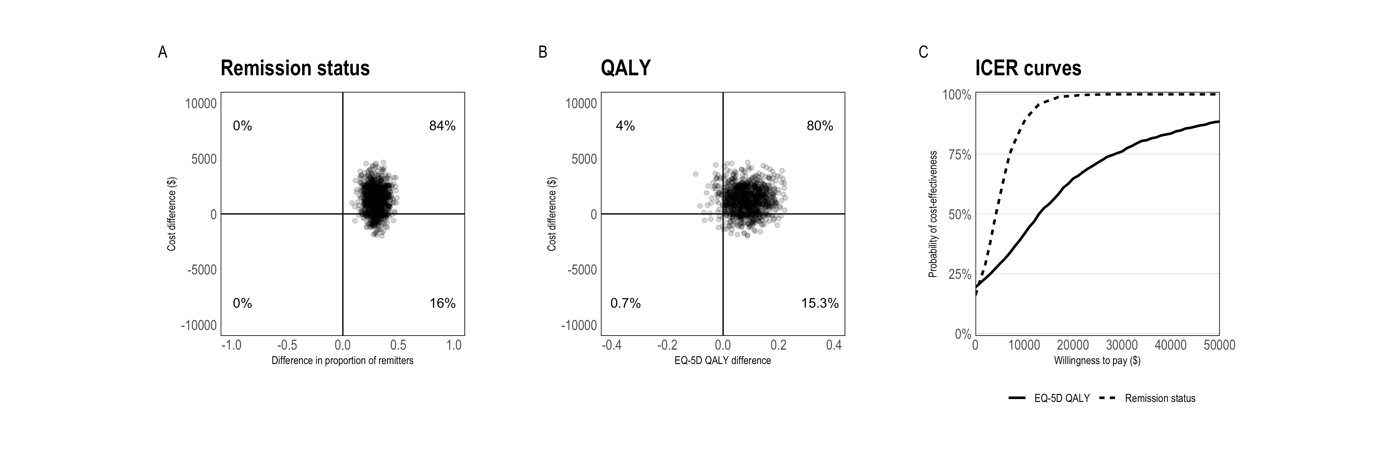
Figure A.6. Pre-treatment to post-treatment cost-effectiveness of BDD-NET compared to supportive therapy (complete cases only).


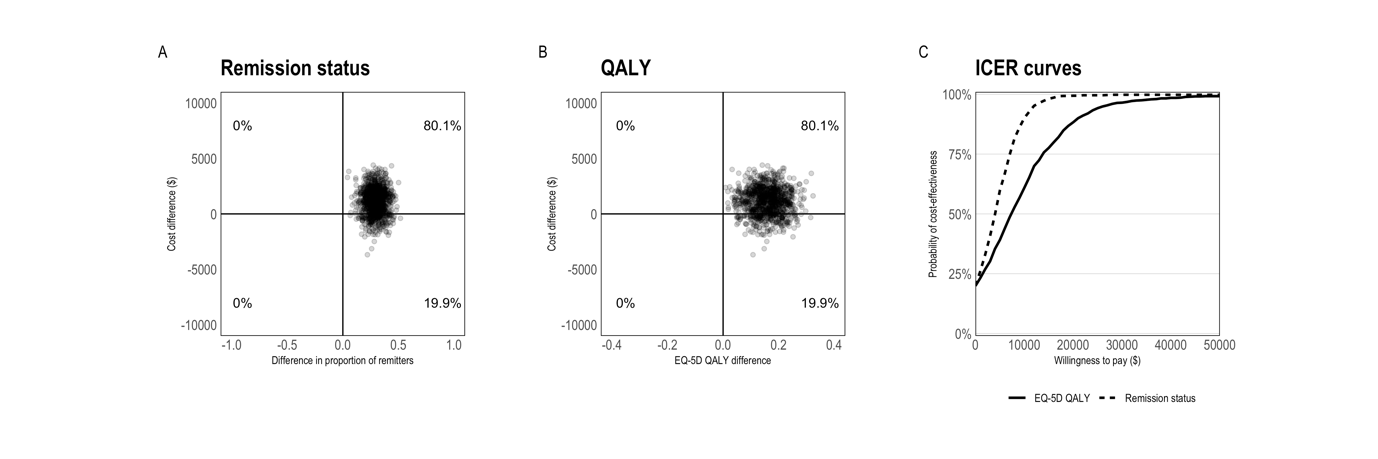


Figure A.7. Pre-treatment to follow-up cost-effectiveness of BDD-NET compared to supportive therapy (complete cases only).
